# Demographic and socio-economic factors affecting bed net ownership, usage, and malaria transmission among adult patients seeking healthcare in two Ghanaian urban cities

**DOI:** 10.1186/s12889-023-17590-8

**Published:** 2024-01-06

**Authors:** Ellis Kobina Paintsil, Enoch Boadi, Anthony Dwamena, Bless Hayford Addo, Agyei Kumi, Kwasi Obiri-Danso, Linda Aurelia Ofori

**Affiliations:** 1 Kumasi Centre for Collaborative Research in Tropical Medicine (KCCR), Kumasi, Ghana; 2 Department of Theoretical and Applied Biology, Kwame Nkrumah University of Science and Technology, Kumasi, Ghana; 3 Department of Molecular Medicine, School of Medical Sciences, Kwame Nkrumah University of Science and Technology, Kumasi, Ghana; 4 Bremang Seventh-Day Adventist Hospital, Suame Municipal, Ghana; 5 Department of Medical Diagnostics, Faculty of Allied Health Sciences, School of Medical Sciences, Kwame Nkrumah University of Science and Technology, Kumasi, Ghana; 6 Sunyani Municipal Hospital, Sunyani Municipal, Ghana

**Keywords:** Bed net, Malaria, Socio-economic, Adult, Urban, Ghana

## Abstract

**Background:**

The most cost-effective malaria prevention and control strategy is the use of a bed net. However, several factors affect the ownership and usage of bed nets among the adult population. Hence, this study aimed to examine socio-demographic factors affecting bed net ownership, usage and malaria transmission among adult patients seeking healthcare in two Ghanaian urban cities.

**Methods:**

This hospital-based cross-sectional study was conducted, between January and September 2021, at Bremang Seventh-Day Adventist Hospital, Suame Municipal, Ashanti Region and Sunyani Municipal Hospital, Sunyani, Bono Region, Ghana. Structured questionnaires were administered to a total of 550 participants to ascertain their ownership and usage of the bed nets. Afterwards, finger prick blood samples were collected for malaria microscopy. Crude and adjusted prevalence ratios (PR) and their respective 95% CIs were calculated, using Poisson regression with robust standard errors, to show associated variables in bivariate and multivariate analyses respectively. R software (version 4.1.1) was used to perform all statistical analyses.

**Results:**

About 53.3% (*n* = 293) of participants owned at least one-bed net but only 21.5% (*n* = 118) slept under it the previous night. Those married were 2.0 (95% CI: 1.6 – 2.5) and 2.4 (95% CI: 1.6 – 3.5) times more likely to own and use a bed net respectively than those who never married. Also, pregnant women were 1.3 (95% CI: 1.1 – 1.6) and 1.8 (95% CI: 1.3 – 2.5) times more likely to own and use a bed net respectively than non-pregnant. Even though income levels were not associated with bed net ownership and usage, students were 0.4 (95% CI: 0.2 – 0.6) and 0.2 (95% CI: 0.1 – 0.5) times less likely to own and use bed net respectively compared to formally employed persons. The overall malaria prevalence rate was 7.8%. Malaria-negative patients were 1.6 (95% CI: 1.2 – 2.0) and 2.4 (95% CI: 1.4 – 4.1) times more likely to own and use bed nets respectively than malaria positive. Patients with tertiary education recorded the lowest malaria prevalence (3.5%, *n* = 4). None of those with a monthly income > $300 recorded a case of malaria. On the contrary, majority 83%, *n*/*N* = 25/30) of the malaria-positive patients earned ≤ $150.

**Conclusion:**

The National Malaria Control Program should conduct comprehensive mapping of all urban population segments before launching mass bed net distribution campaigns, taking into account demographic and socioeconomic factors to enhance bed net utilization and reduce malaria prevalence.

## Introduction

Ghana records about 2% and 3% of global malaria cases and deaths respectively, making it part of the 15 countries in the world with the heaviest malaria burden [1]. Although most malaria cases and deaths occur in children, a considerable number of adults also get infected and experience the associated morbidity and mortality [2–4]. Infected adults may serve as a reservoir for vulnerable groups such as children under five years and pregnant women by carrying low-density parasites for long periods [5]. It has been debated for decades as to whether malaria is a consequence of or a cause of poverty [6]. Nonetheless, there is a strong consensus that its impact is ferocious especially on people with low socioeconomic status as they are least able to afford medical treatment and preventive measures [7].

Among the various malaria prevention and control strategies implemented in endemic areas, available evidence suggests that the use of a bed net is the most cost-effective [8]. For this reason, Ghana's National Malaria Control Program (NMCP) aims to embark on a mass distribution campaign every other year to promote ownership and use of Insecticide Treated Nets (ITNs) [9]. However, it is unable to embark on such campaigns regularly due to the huge cost involved [9]. The success of such mass distribution campaigns when implemented varies from region to region due to limited staffing, logistical challenges and the short time required to complete the campaign [10]. For effective protection against malaria by bed nets, the WHO recommends that at least 80% of household members should have access to ITNs [11]. Even the use of an untreated bed net provided it is in relatively good condition can reduce malaria transmission [12, 13]. High household ownership of bed nets immediately after a successful mass distribution campaign turns to decline within two years as some nets are lost or damaged [14]. Also, high bed net ownership does not automatically lead to high utilization [15]. The self-reported reasons for not utilizing a bed net when available include discomfort due to heat, the smell of the net and difficulty in hanging [16].

The main objective of the bed net distribution initiative is to prevent transmission among vulnerable groups such as poor people living in rural areas, pregnant women and children under five years [17, 18]. Hence, there is less free bed net available for urban households with non-pregnant women and/or without children residing in them. The situation could worsen if households are unable to purchase bed nets from the open market due to their low socio-economic status [19]. Nonetheless, adults living in urban areas are prone to malaria [20], therefore they must be considered when designing and implementing malaria control strategies. Although some previous studies have reported on the relationship between socio-demographic factors and bed net ownership, usage and malaria transmission among vulnerable sub-populations [4, 17, 21, 22], such information is lacking among the urban adult population. This paper seeks to examine socio-economic and demographic factors affecting bed net ownership, usage and malaria transmission among adult patients seeking healthcare in two Ghanaian urban cities.

## Materials and methods

### Ethical considerations

Ethics approval was obtained from the Committee on Human Research, Publication and Ethics at the Kwame Nkrumah University of Science and Technology, Kumasi, Ghana (Protocol Number-CHRPE/AP/612/19). Study participants who could read and understand the study protocol provided written informed consent after receiving adequate information about the study. In the case of participants with no formal education hence could not read and understand, the study protocol was explained to them in their local language and informed consent was obtained from their legal guardian(s).

### Study area

**Fig. 1:**
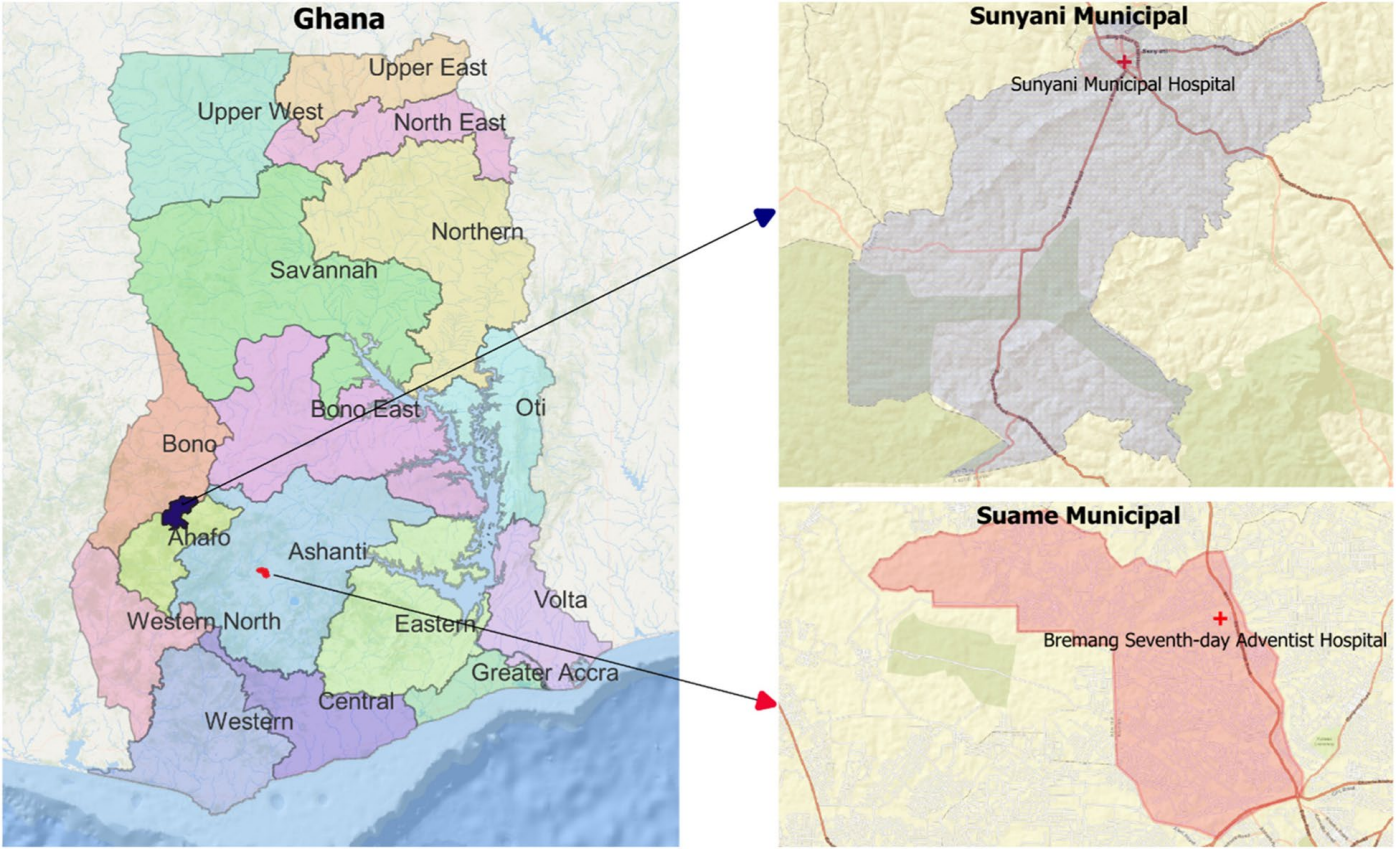
Map of Ghana showing the two urban towns and hospitals that participants were recruited from

This study was conducted at Bremang Seventh-Day Adventist Hospital in Suame Municipal, Ashanti Region and Sunyani Municipal Hospital in Sunyani, Bono Region. These two healthcare institutions were chosen due to their status as the primary healthcare providers for the majority of adults residing within their respective catchment areas. The last mass distribution campaign of INTs in both study areas occurred in 2019, two years prior to the start of this study. Suame Municipal is an urban town with an estimated adult (≥ 18 years old) population of 81,774 [23] and shares boundaries with Kumasi Metropolitan, the capital town of the Ashanti Region (Fig. 1). Sunyani is the capital town of the Bono Region with an estimated adult (≥ 18 years old) population of 116,157 [23]. Sunyani Municipal Hospital is a primary healthcare facility specialized in providing quality healthcare to clients in and around Sunyani (Fig. 1). The working population of the selected cities is mainly made up of people belonging to the middle class with the majority of them depending on small to medium business ventures as a source of livelihood.

### Study design

A hospital-based cross-sectional study, was conducted between January and September 2021. Convenience sampling was employed to select adults seeking healthcare at the Outpatient Departments (OPD) of the two selected hospitals. After participants gave their informed consent, a structured questionnaire was administered to collect data. The questionnaire was initially pretested and difficult-to-understand questions were rephrased for easy understanding. The questionnaire collected data on demographic characteristics (sex, age, ethnicity and marital status), socioeconomic status (education, occupation, income and house ownership) and bed net ownership and usage. Bed net usage was defined as sleeping under a bed net the previous night preceding the study. After administering the questionnaire, finger prick blood samples were collected from all participants for thick and thin blood smears.

This study included patients visiting the outpatient departments of the two health facilities during the study period. Patients who were too ill to speak and/or needed hospital admission and those who were already admitted were excluded.

### Sample size

The minimum sample size was determined using data from a recent nationwide survey, which indicated a 73% prevalence of bed net ownership in Ghanaian households [9]. Based on the population of the two study sites (≈200,000) [23], it was established that a minimum sample size of 303 individuals would achieve a 95% confidence interval with a margin of error of 5% and 80% statistical power. However, the study ultimately enrolled a total of 550 adult participants.

### Malaria microscopy

Malaria microscopy was done according to the procedure described by Haggaz et al. [24]. In summary, thin and thick blood smears were prepared on the same microscope slide using approximately 2 μl and 6 μl of capillary blood respectively and stained with 10% Giemsa for 10 min. Afterwards, each slide was observed under the × 100 oil immersion objective lens of a light microscope by two independent and experienced laboratory scientists who were blinded to each other's results. The thick smears were evaluated for the presence of *Plasmodium* parasites. At least 100 high-power fields had to be examined before a smear was considered negative. Parasite densities were recorded as a ratio of parasites to 200 WBC in thick films (or to 500 WBC, if the parasite count was less than 100 per 200 WBC), assuming a leukocyte count of 8000 cells/µl. When more than 300 parasites were seen per high power field on the thick film, then a thin blood smear was used for parasite estimation. The species identification of all positive thick smears was done using their corresponding thin smears. When disagreement occurred, a third independent reader was employed. All microscopists had a level A expertise according to the National Competency Assessment in Malaria Microscopy (NCAMM). Parasite densities (parasite/μl of whole blood) were calculated using the assumed WBC counts of 8.0 × 10^9^/L.

$$\mathrm{Parasite\,densities\,}=\,(\mathrm{Number\,of\,parasites\,counted}/\mathrm{WBC\,counted})\,\times\,\mathrm{ Assumed\,WBC\,counts}.$$

### Variables and operational definition

The responses to the questionnaire and the laboratory results of the malaria test were entered into Microsoft Excel 2019.

### Dependent variables

Bed net ownership (owning at least one bed net): yes/no.

Bed net usage (sleeping under a bed net the previous night preceding the study): yes/no.

Having malaria (malarial parasite diagnosis was established by smear microscopy): positive/negative.

### Independent variable

Sex: male/female.

Age: in years.

Ethnicity: Akan; Mole Dagbani; other (specify).

Marital status: married; never married; cohabiting; other (specify).

Religion: Christian; Muslim; other (specify).

Formal Education: formal educational level was defined as the highest completed education, which was categorized into four groups: none; primary; secondary; tertiary.

Occupation: divided into four categories: informally employed; formally employed; unemployed; student.

Monthly income: in dollars: ≤ 150; 151 – 300; > 300.

House ownership: renting; owning.

### Data analysis

Descriptive statistics for sociodemographic variables and bed net ownership and usage were done using absolute frequencies and their corresponding percentages. Crude prevalence ratio (PR) and adjusted prevalence ratios (APR), and their respective 95% CIs were calculated, using Poisson regression with robust standard errors, to show associated variables in bivariate and multivariate analyses respectively [25]. The dependent variables were own or do not own bed net; used or do not use a bed net and malaria positive or negative. Demographic and socio-economic characteristics were the independent variables considered in the regression analysis. The geometric mean of parasite density and their corresponding 95% confidence interval (CI) were used to calculate the population measures of malaria parasitaemia. Because of the exploratory nature of this study, no *p*-values were calculated; instead, confidence intervals (CIs) were used [26]. R software (version 4.1.1) was used to perform all statistical analyses [27]. The sandwich package (version 3.0–0) was used to compute robust estimators of the Poisson regression. The figure which shows the prevalence of bed net ownership, usage and malaria transmission among pregnant and non-pregnant women and males was plotted using *ggplot2* package (version 3.3.5). QGIS software, version 3.24.0 [28] was used to draw a map to show the geographical location of the two study sites.

## Results

### Socio-economic and demographic characteristics of study participants

In Table 1, the socio-economic and demographic characteristics of study participants are presented. Out of the 550 participants, 332 (60.4%) were from Suame and 218 (39.6%) from Sunyani. Females accounted for most (76.5%, *n*/*N* = 421/550) of the study participants. Also, more than half of the participants (52.5%, *n*/*N* = 289/550) were between the ages of 18 – 30. While 49.5% (*n*/*N* = 272/550) of the participants were married, 37.6%, (*n*/*N* = 207/550) had never married at the time of the survey. About 20.9% (*n*/*N* = 115/550) had attained a tertiary level of education while those with no education were only 4.7% (*n*/*N* = 26/550). Most (68.7%, *n*/*N* = 378/550) of the participants were either formally or informally employed with 17.5% (*n*/*N* = 96/550) being unemployed. Only 4.0% (*n*/*N* = 15/378) of those employed earn more than $300 per month with the majority (78.3%, *n*/*N* = 294/378) earning $150 or less. About 47.1% (*n*/*N* = 259/550) of the study participants lived in a house owned by their household head.

**Table 1:** Socio-economic and demographic characteristics of study participants

	**Suame, n (%)**	**Sunyani, n (%)**	**Total, n (%)**
**Sex**			
Female	245 (73.8)	176 (80.7)	421 (76.5)
Male	87 (26.2)	42 (19.3)	129 (23.5)
**Age**			
18 – 30	180 (54.2)	109 (50.0)	289 (52.5)
31 – 40	82 (24.7)	48 (22.0)	130 (23.6)
41 – 50	37 (11.2)	25 (11.5)	62 (11.3)
51 +	33 (9.9)	36 (16.5)	69 (12.6)
**Ethnicity**			
Akan	296 (89.2)	141 (64.7)	437 (79.5)
Mole Dagbani	30 (9.0)	70 (32.1)	100 (18.1)
Other	6 (1.8)	7 (3.2)	13 (2.4)
**Marital status**			
Married	158 (47.6)	114 (52.3)	272 (49.5)
Never married	126 (38.0)	81 (37.2)	207 (37.6)
Cohabiting	34 (10.2)	3 (1.4)	37 (6.7)
Other	14 (4.2)	20 (9.1)	34 (6.2)
**Religion**			
Christians	311 (93.7)	183 (83.9)	494 (89.8)
Muslims	21 (6.3)	35 (16.1)	56 (10.2)
**Formal Education**			
None	12 (3.6)	14 (6.4)	26 (4.7)
Primary	129 (38.9)	99 (45.4)	228 (41.5)
Secondary	123 (37.0)	58 (26.6)	181 (32.9)
Tertiary	68 (20.5)	47 (21.6)	115 (20.9)
**Occupation**			
Informally employed	185 (55.7)	111 (50.9)	296 (53.8)
Formally employed	45 (13.5)	37 (17.0)	82 (14.9)
Unemployed	56 (16.9)	40 (18.3)	96 (17.5)
Student	46 (13.9)	30 (13.8)	76 (13.8)
**Monthly income ($)**^**a**^			
≤ 150	177 (77.0)	117 (79.1)	294 (77.8)
151 – 300	45 (19.5)	24 (16.2)	69 (18.2)
> 300	8 (3.5)	7 (4.7)	15 (4.0)
**House ownership**			
Renting	172 (51.8)	119 (54.6)	291 (52.9)
Owning	160 (48.2)	99 (45.4)	259 (47.1)

^a^for only those who were employed

A slight majority (53.3%, *n*/*N* = 293/550) of the study participants owned at least one bed net; however, only 21.5% (*n*/*N* = 118/550) slept under it the night before the survey. Malaria-negative individuals were 1.6 (95% CI: 1.2 – 2.0) and 2.4 (95% CI: 1.4 – 4.1) times more likely to own and use bed nets respectively than malaria-positive patients. The ownership and usage of the bed net varied by study site. More participants (64.7%, *n*/*N* = 141/218) from Sunyani than Suame (45.8%, *n*/*N* = 152/332) own a bed net (APR = 1.4; 95% CI: 1.2 – 1.6). The analysis of adjusted prevalence ratios (APR) reveals that individuals subjects from Sunyani were 1.8 (95% CI: 1.3 – 2.5) times more likely to use a bed net than their counterparts from Suame. More females owned (APR = 1.5; 95% CI: 1.2 – 1.9) and used (APR = 1.8; 95% CI: 1.3 – 3.0) bed net than males. Among the various age groups, the prevalence of bed net use was highest (APR = 1.9, 95% CI: 1.3 – 2.7) in the 31 – 40 years age group when compared to the reference age group of 18 – 30 years. Based on the APR, bed net ownership and usage were similar among the different ethnic groups studied. However, those married were 2.0 (95% CI: 1.6 – 2.5) and 2.4 (95% CI: 1.6 – 3.5) times more likely to own and use a bed net respectively than those who had never married.

The ownership of bed net was 0.7 (95% CI: 0.6 – 0.8) times lower among participants with secondary school education and above compared to those with primary education and below. Nonetheless, these two groups equally used bed net (APR = 0.8; 95% CI: 0.5 – 1.1). Ownership of more than two-bed nets per household was not associated with higher usage when compared with households who owned two or less (APR = 1.0, 95% CI: 0.7 – 1.5). Those informally employed (APR = 1.0; 95%, CI: 0.8 – 1.3) or unemployed (APR = 1.0; 95% CI: 0.8 – 1.3) had similar prevalence of bed net ownership compared to formally employed participants. However, students were 0.4 (95% CI: 0.2 – 0.6) and 0.2 (95% CI: 0.1 – 0.5) times less likely to own and use bed net respectively compared to formally employed persons. Income levels were not associated with bed net ownership and usage. Nonetheless, those who lived in a house owned by their household head recorded a higher prevalence of bed net usage than their counterpart living in a rented apartment (APR = 1.3, 95% CI: 1.1 – 1.8). The crude and adjusted prevalence ratios of socio-economic and demographic characteristics associated with bed net ownership and usage are further presented in Table 2.

**Table 2:** Socio-economic and demographic associations with the probability of bed net ownership and usage among the study participants

Variable	Bed net ownership		Bed net usage	
	**PR (95% CI)**	**APR (95% CI)**	**PR (95% CI)**	**APR (95% CI)**
**Malaria status**				
**Positive**	1			
**Negative**	**1.6 (1.2 – 2.0)**	**1.6 (1.2 – 2.0)**	**2.4 (1.4 –4.1)**	**2.4 (1.4 –4.1)**
**Location**				
Kumasi	1			
Suame	**1.4 (1.2 – 1.6)**	**1.4 (1.2 – 1.6)**	**1.9 (1.4 – 2.6)**	**1.8 (1.3 – 2.5)**
**Sex**				
Male	1			
Female	**1.5 (1.2 – 1.9)**	**1.5 (1.2 – 1.9)**	**2.0 (1.2 – 3.2)**	**1.8 (1.3 – 3.0)**
**Age**				
18 – 30	1			
31 – 40	**1.6 (1.3 – 1.9)**	**1.6 (1.3 – 1.9)**	**1.9 (1.3 – 2.7)**	**1.9 (1.3 – 2.7)**
41 – 50	**1.5 (1.3 – 2.0)**	**1.5 (1.2 – 1.9)**	1.5 (0.9 – 2.5)	1.4 (0.9 – 2.4)
≥ 51	**1.5 (1.2 – 1.9)**	**1.4 (1.1 – 1.8)**	1.6 (1.0 – 2.6)	1.5 (0.9 – 2.5)
**Ethnicity**				
Akan	1			
Mole Dagbani	**1.3 (1.1 – 1.6)**	1.1 (0.9 – 1.4)	**1.5 (1.0 – 2.1)**	1.2 (0.8 – 1.7)
Other	1.1 (0.7 – 1.5)	1.0 (0.7 – 1.5)	0.9 (0.4 – 2.2)	0.8 (0.4 – 1.9)
**Marital status**				
Never married	1			
Married	**2.0 (1.7 – 2.5)**	**2.0 (1.6 – 2.5)**	**2.4 (1.6 – 3.5)**	**2.3 (1.6 – 3.5)**
Other	**1.6 (1.2 – 2.2)**	**1.7 (1.7 – 1.4)**	1.3 (0.7 – 2.4)	1.3 (0.7 – 2.5)
**Education**				
Primary and below	1			
Secondary and above	**0.7 (0.6 – 0.8)**	**0.7 (0.6 – 0.8)**	0.7 (0.5 – 1.0)	0.8 (0.5 – 1.1)
**Occupation**				
Formally employed	1			
Informally employed	1.0 (0.8 – 1.3)	1.0 (0.8 – 1.3)	**0.2 (0.1 – 0.3)**	**0.2 (0.1 – 0.4)**
Unemployed	0.9 (0.7 – 1.2)	1.0 (0.8 – 1.3)	0.8 (0.5 – 1.3)	0.8 (0.5 – 1.3)
Student	**0.4 (0.2 – 0.6)**	**0.4 (0.2 – 0.6)**	**0.2 (0.1 – 0.5)**	**0.2 (0.1 – 0.5)**
**Monthly income ($)**^**a**^				
≤ 151	1			
151 – 300	0.9 (0.7 – 1.2)	1.0 (0.7 – 1.2)	1.3 (0.8 – 2.1)	1.4 (0.9 – 2.2)
> 300	0.9 (0.5 – 1.5)	0.8 (0.5 – 1.4)	1.2 (0.4 – 3.1)	1.0 (0.3 – 3.0)
**House ownership**				
Renting	1			
Owning	1.0 (0.9 – 1.2)	1.1 (0.9 – 1.2)	**1.3 (1.1 – 1.8)**	**1.3 (1.1 – 1.8)**
**Household size**				
< 5	1			
≥ 5	0.9 (0.8 – 1.1)	0.9 (0.8 – 1.1)	0.8 (0.5 – 1.1)	0.8 (0.5 – 1.1)

Abbreviations: *PR* Crude prevalence ratio, *APR* Adjusted prevalence ratio, *CI* Confidence interval

^a^for only those who were employed. PR and APR significantly higher or lower than 1 are shown in bold

### Socio-economic risk factors for malaria

The overall malaria prevalence was 7.8% (*n*/*N* = 43/550), the prevalence was similar in Suame (7.5%, *n*/*N* = 25/332) and Sunyani (8.3%, *n/N* = 18/218) (APR = 0.9; 95% CI: 0.5 – 1.6). *Plasmodium falciparum* constituted the majority (97.6%, *n*/*N* = 42/43) of the infections, with *Plasmodium ovale* being responsible for only one (2.3%) case. Fewer females (6.4%, *n*/*N* = 27/421) were positive for malaria compared to males (12.4%, *n*/*N* = 16/129) (APR = 0.4; 95% CI: 0.3 – 0.8). There were no statistically significant differences in the number of malaria-positive cases recorded by adults aged 18–30 when compared to those in the age groups 31–40 (APR = 0.6; 95% CI: 0.2 – 1.7), 41–50 (APR = 0.4; 95% CI: 0.2 – 1.5), and ≥ 51 (APR = 0.8; 95% CI: 0.4 – 2.2).

The relationship between malaria infection and socio-economic variables is displayed in Table 3. Significant differences in malaria prevalence were observed only between males (12.4%, *n*/*N* = 16/129) compared to females (6.4%, *n*/*N* = 27/421) and those with tertiary education (3.5%, *n*/*N* = 4/115) compared to those with no formal education (15.4%, *n*/*N* = 4/26). For all other variables, no significant differences in malaria prevalence were found. Nonetheless, the prevalence of malaria varied based on the nature of their occupation. Students (13.2%, *n*/*N* = 10/76) followed by those informally employed (8.4%, *n*/*N* = 25/296) recorded the highest infection rate. Among participants who were employed, none of those earning a monthly income > $300 recorded a case of malaria; contrary, the majority 83%, *n*/*N* = 25/30) of the malaria-positive patients earned ≤ $150.

**Table 3:** Malaria parasite prevalence in relation to socio-economic variables

Variable	Prevalence (n/N)	PR (95% CI)	APR (95% CI)
**Malaria**	7.8 (43/550)	NA	NA
**Malaria species**			
*Plasmodium falciparum*	97.6 (42/43)	NA	NA
*Plasmodium ovale*	2.3 (1/43)	NA	NA
**Sex**			
Male	12.4 (16/129)	1	
Female	6.4 (27/421)	**0.5 (0.3–0.9)**	**0.4 (0.3–0.8)**
**Age**			
18 – 30	9.3 (27/289)	1	1
31 – 40	5.4 (7/130)	0.6 (0.3–1.3)	0.6 (0.2–1.7)
41 – 50	4.8 (3/62)	0.5 (0.2–1.7)	0.4 (0.2–1.5)
≥ 51	8.7 (6/69)	0.9 (0.4–2.2)	0.8 (0.4–2.2)
**Educational level**			
None	15.4 (4/26)	1	1
Primary	7.0 (16/228)	0.5 (0.2–1.3)	0.4 (0.2–1.2)
Secondary	10.5 (19/181)	0.7 (0.3–1.9)	0.8 (0.3–2.0)
Tertiary	3.5 (4/115)	**0.2 (0.1–0.9)**	**0.2 (0.2–0.9)**
**Occupation**			
Formally employed	6.1 (5/82)	1	1
Informally employed	8.4 (25/296)	1.4 (0.6–3.5)	1.2 (0.5–3.1)
Unemployed	3.1 (3/96)	0.5 (0.1–2.1)	0.7 (0.3–2.4)
Student	13.2 (10/76)	2.2 (0.8–6.0)	2.2 (0.8–6.0)
**Monthly income ($)**^**a**^			
≤ 150	8.5 (25/294)	1	1
151 – 300	5.2 (5/69)	0.9 (0.3–2.2)	0.9 (0.3–2.2)
> 300	0 (0/15)	NA	NA
**House ownership**			
Rented	7.8 (23/294)	1	1
Household head	8.5 (20/236)	1.1 (0.6–1.9)	1.3 (0.6–2.1)

Abbreviations: *PR* Crude prevalence ratio, *APR* Adjusted prevalence ratio, *CI* Confidence interval

^a^for only those who were employed; *NA* Non-applicable. PR and APR significantly higher or lower than 1 are shown in bold

Table 4 presents the geometric mean parasite density in malaria positive participants based on various socio-economic variables. Regarding gender, males had a slightly higher mean parasite density (5726/µL, 95% CI: 3446 – 9513) compared to females (4853/µL, 95% CI: 3243 – 7265). Age-wise, individuals aged ≥ 51 exhibited the highest parasite density (11,867/µL, 95% CI: 3876 – 36,334). Education level showed notable differences, with those having only primary education displaying the highest mean parasite density (12,449/µL, 95% CI: 6983 – 22,196). The occupation category demonstrated differences as well, with formally employed individuals having a lower parasite density (2175/µL, 95% CI: 1049 – 4511) than informally employed (6066/µL, 95% CI: 3951 – 9313). Monthly income and house ownership also displayed varying parasite densities among the subgroups.

**Table 4:** Malaria parasite density in relation to socio-economic variables

Socio-economic variable	Geometric mean parasite density (95% CI)/µL of blood
**Sex**	
Male	5726 (3446 – 9513)
Female	4853 (3243 – 7265)
**Age**	
18 – 30	4313 (2989 – 6223)
31 – 40	6122 (2921 – 12838)
41 – 50	3300 (658 – 16562)
≥ 51	11867 (3876 – 36334)
**Educational level**	
None	827 (349 – 1960)
Primary	12449 (6983 – 22196)
Secondary	5952 (3933 – 9007)
Tertiary	2028 (794 – 5180)
**Occupation**	
Formally employed	2175 (1049 – 4511)
Informally employed	6066 (3951 – 9313)
Unemployed	2285 (390 – 13384)
Student	6780 (3824 – 12022)
**Monthly income ($)**^**a**^	
≤ 150	5820 (3516 – 9634)
151 – 300	2694 (1130 – 6420)
> 300	NA
**House ownership**	
Rented	3378 (2355 – 4844)
Household head	8143 (4777—13881)

Abbreviations: *NA* Non-applicable

^a^for only those who were employed

### Malaria prevalence and bed net ownership and usage among pregnant women

**Fig. 2:**
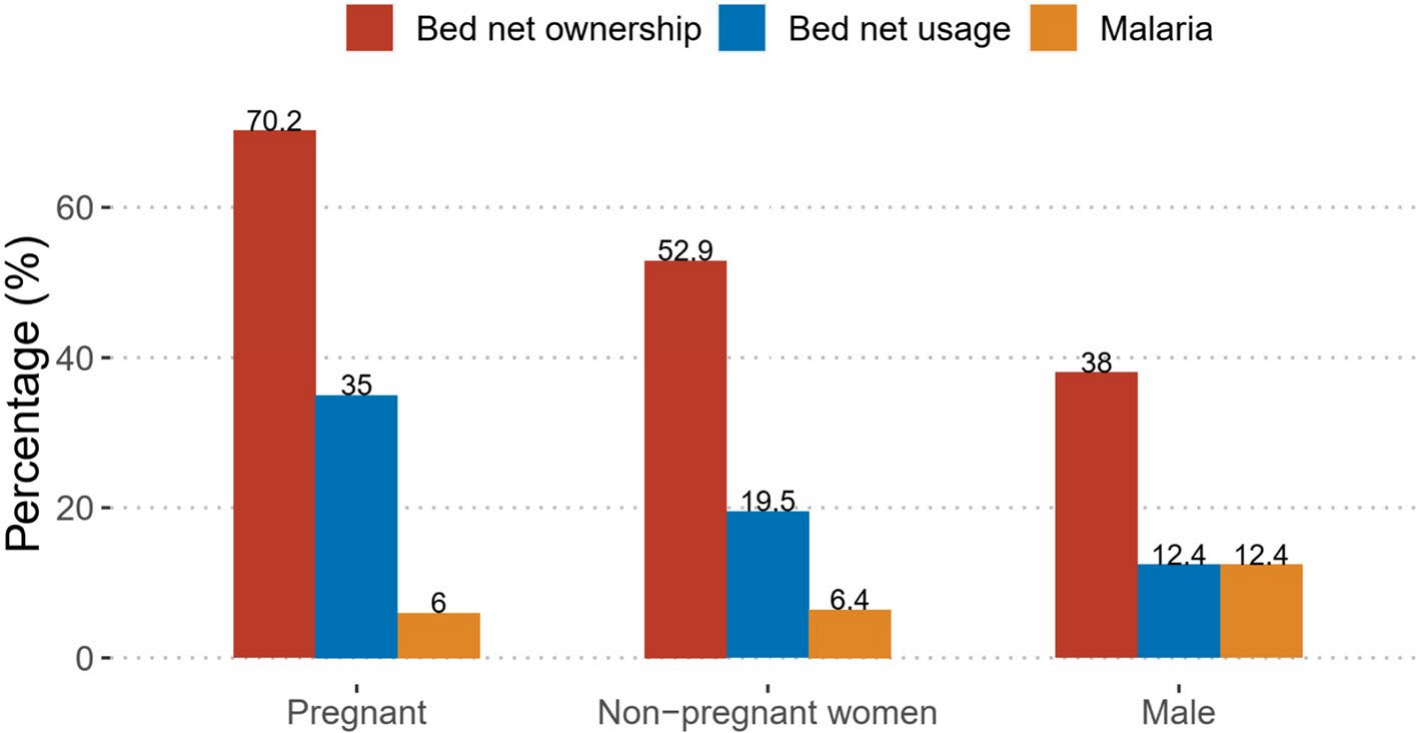
Prevalence of bed net ownership, usage and malaria transmission among pregnant and non-pregnant women and males

Out of the 421 female participants that were sampled, 124 (29.5%) were pregnant with an age range of 18 – 49 years. Figure 2 shows the prevalence of bed net ownership, usage and malaria prevalence among pregnant and non-pregnant women and males. Pregnant women were 1.3 (95% CI: 1.1 – 1.6) and 1.8 (95% CI: 1.3 – 2.5) times more likely to own and use a bed net respectively than non-pregnant patients. However, the prevalence of malaria infection in pregnant women and non-pregnant individuals was similar (APR = 0.8; 95% CI: 0.4 – 1.7).

## Discussion

Our results show that bed net ownership (53.3%) in households of adults attending the hospitals sampled in the two cities was lower than the proposed universal coverage mark of 80% and above [11]. The current bed net ownership rate is lower than the results from a 2016 nationwide survey which reported that 73% of households in Ghana own at least one ITN [9]. Although the last mass distribution campaign of INTs in both study areas took place in 2019, Suame which shares a boundary with a larger city, Kumasi, recorded lower bed net ownership and usage when compared to Sunyani. Other studies conducted in households with vulnerable smaller towns and rural areas have reported bed net ownership closer to the universal coverage mark [17, 21]. Urban areas have high population density which is usually heterogeneous in terms of ethnicity, religion, occupation and socioeconomic background. Hence, mass bed net distribution campaigns in bigger cities are likely to face challenges such as incomplete mapping of the population and low patronage. This could explain the disparities in bed net ownership and usage observed among the two cities in the current study and other earlier studies conducted in rural areas.

Nonetheless, among pregnant women, we reported high bed net ownership comparable to the universal coverage mark. Also, pregnant women were more likely to use bed nets compared to non-pregnant women. These women are usually given bed nets for free or at a subsidised cost during the antenatal clinic (ANC) [29] and educated on the benefits of using them [30, 31]. The reason why not all pregnant women who own bed net use it could be difficulty in hanging the net, discomfort resulting from the heat and smell of the net [16]. The frequency of bed net ownership and usage was higher among married participants than never married. Married couples are more likely to have children, making them eligible for additional nets during pregnancy and when their children get vaccinated thus increasing the number of available nets in the household. The education received on the benefits of bed nets during pregnancy and vaccination of children and also the responsibility of protecting other family members against infections could explain the high usage of bed nets among this group [32].

The findings of this study showed that ownership and use of bed net in the city setting is influenced by some socio-economic variables. Those with a secondary school level of education and above were less likely to own a bed net; however, educational level did not affect bed net utilization. Similarly, a recent study did not find significant disparities in bed net utilization by educational status [17]. This could be explained by the fact that formal education offers limited information on malaria control strategies compared to the information received during health campaigns or hospital visits [31]. The current study and a similar one conducted in Cameroon [31] showed no association between income and bed net ownership and utilisation. Inequities in bed net ownership are decreased by free mass-distribution campaigns. During such campaigns, the poor and least educated are the ones who mostly avail themselves [29], since middle-income Ghanaians prefer and are willing to pay the commercial price for bed net with certain design features that meet their preferences [33, 34]. We found that compared to those who were formally employed, adult students were less likely to own and use a bed net. Similarly, a study conducted in Nigeria reported that the majority of Secondary school students knew about bed nets; nonetheless, only less than half use them [35]. The low perception of risk from malaria among the adult student population could account for the low ownership and usage of bed nets observed [36]. Individuals who perceive malaria infection to be less fatal and believe that bed nets are not an effective means of preventing the infection are less likely to own a net [36]. In addition, some students who miss out on the free mass-distribution campaigns may be unable to afford a commercially available bed net because of the high cost and their low-income levels.

The overall malaria prevalence of 7.8% recorded by our study shows that malaria is still a public health concern among the adult population seeking healthcare in the two cities sampled. Studies conducted among a similar population in Ethiopia [2] and Uganda [37] recorded comparable prevalence. However, other studies have reported a higher (≥ 14%) frequency of malaria in the adult population in Ghana [3, 4], Nigeria [20, 38] and Kenya [39]. This variation might be due to geographical differences; since malaria is more prevalent in rural areas [9]. Also, one study that recorded a higher prevalence was conducted only on suspected febrile patients [38]. *Plasmodium falciparum* was the dominant *Plasmodium* species responsible for the majority of the infections, this finding is in line with several studies conducted in Africa [2, 4, 22, 38, 39].

We observed that those who own or used bed nets were less likely to be infected with malaria. The current finding is in agreement with several other studies which also observed significantly lower malaria prevalence among participants who used bed nets [40–42]. Even though we found that ownership of bed nets was associated with lower infection, an earlier study had reported that ITN campaigns which often lead to increased ownership do not reduce malaria prevalence over time since utilization turn to decrease with years of ownership [19]. Also, our study suggests that lack of education and low income were associated with an increased prevalence of malaria. Similarly, other studies have linked low educational status [7, 43, 44] and income [7, 45] with high malaria prevalence. Indeed, education and income increase one’s ability to access and implement health-related guidelines. High-income earners tend to live in nice neighbourhoods with lower risk of malaria transmission they also have the ability to buy insecticide sprays and insecticide-treated nets (ITNs) which may help to decrease their contact with mosquito vectors.

Our study had some limitations. Due to the cross-sectional nature of the study, sampling was not continuously done throughout the year hence we were unable to show seasonal relationships. This study did not differentiate between the different types of bed nets such as untreated bed nets, ITNs and long-lasting insecticide nets (LLINs) owned and used by participants, nor did it investigate their sources of supply. There were no independent observations carried out to validate the responses on bed net usage and socio-economic status provided by participants. Also, because of the exploratory nature of this study, our findings apply solely to the studied population. Irrespective of the aforementioned limitations, our study identified socio-economic factors associated with bed net ownership, utilization and malaria transmission among adult patients seeking healthcare in two Ghanaian urban cities which could provide a foundation for more in-depth research.

## Conclusion

Despite malaria being endemic in the urban adult population in Ghana, bed net coverage and usage which serves as a malaria prevention and control strategy remains low. Bed net coverage and usage were relatively high among pregnant women but low among students. Hence, bed net distribution campaigns and education on the benefits of using it should not only be limited to the ANC but outpatient departments (OPD) and adult students as well. Age, marital status, educational level and type of occupation were related to bed net ownership and usage. Similarly, bed net usage, educational status and income were associated with malaria infection in the population. Hence, the National Malaria Control Program should conduct comprehensive mapping of all urban population segments before launching mass bed net distribution campaigns, taking into account demographic and socioeconomic factors to enhance bed net utilization and reduce malaria prevalence.

## Data Availability

The data supporting the findings of this study are available from the corresponding author upon request.
